# Analysis of the potential profile and influencing factors of health behavior protection motivation in patients with unruptured intracranial aneurysms based on protection motivation theory

**DOI:** 10.3389/fpubh.2026.1942155

**Published:** 2026-10-14

**Authors:** Shuyuan Huang, Yidan Liu, Wenguang Xie, Chao Zhang, Xingru Li, Shilin Liu, Wei Xiao, Wenhao Zhang, Ping Zhou

**Affiliations:** 1 Department of Nursing, The Second Affiliated Hospital of Nanchang University, Nanchang, China; 2 School of Nursing, Jiangxi Medical College of Nanchang University, Nanchang, China

**Keywords:** health behavior, influencing factors, latent profile analysis, theory of protective motivation, unruptured intracranial aneurysm

## Abstract

**Background:**

Rupture of unruptured intracranial aneurysms (UIA) is the third leading cause of morbidity and mortality from cerebrovascular disease, with a disability rate as high as 36–58%; health behaviors are crucial for reducing the risk of rupture. Protection Motivation Theory serves as an effective framework for explaining the formation of health behaviors; however, there is currently a lack of analyses on the latent categories of protection motivation for health behaviors among UIA patients both domestically and internationally, and in-depth exploration of population heterogeneity and intrinsic motivation has been neglected. Based on Protection Motivation Theory, this study explores the overall level and dimensional characteristics of protection motivation for health behaviors among UIA patients, analyzes its latent profiles and influencing factors, and provides a reference for clinical stratified management and personalized interventions.

**Methods:**

From July to December 2025, a convenience sample of 300 UIA patients meeting the inclusion criteria was recruited from a Grade A tertiary hospital in Jiangxi Province for a clinical survey. A self-designed general demographic questionnaire, the UIA Patient Health Behavior Protective Motivation Scale, and the Health Promotion Lifestyle Scale II (HPLP-II) were used to assess the current status of health behavior protective motivation, health-promoting lifestyles, and their relationship among UIA patients. This study employed latent profile analysis (LPA) to identify latent profiles among UIA patients. One-way analysis of variance (ANOVA) was used to compare differences in general demographic data and disease-related scores across latent profiles. Chi-square tests and Fisher’s exact test were used for categorical variables. Pearson correlation analysis was used to examine the relationship between protection motivation and health-promoting behaviors. Multinomial logistic regression was used to investigate the factors influencing these latent profiles.

**Results:**

The results of this study showed that the total score for health behavior protection motivation among UIA patients was (81.96 ± 15.41) points, and the total score for health behavior promotion was (126.83 ± 30.78) points. The total score for health behavior protection motivation was positively correlated with the total scores of the HPLP-II and the HPLP-II (*r* = 0.73, *p* < 0.001),and the total scores of each dimension were positively correlated with the total score for health behavior promotion (*p* < 0.001). One-way ANOVA revealed significant differences in HPLP-II total scores across the three latent profiles (*F*(2, 297) = 136.459, *p* < 0.001). Chi-square tests showed significant differences in educational level, sleep quality, smoking, alcohol consumption, and comorbid chronic diseases across the three groups (*χ*^2^ = 7.693–60.171, df = 2–4, all *p* < 0.05). This study identified three latent profiles of protective motivation: the low protective motivation group (21.0%), the moderate protective motivation group (53.0%), and the high protective motivation group (26.0%). Multinomial logistic regression analysis showed that educational level (OR = 6.997, 95% CI: 2.053–23.850), sleep quality (OR = 5.069, 95% CI: 1.644–15.626), and HPLP-II total score (OR = 1.078, 95% CI: 1.051–1.105) were facilitators for the moderate group, while smoking (OR = 0.357, 95% CI: 0.138–0.928) and alcohol consumption (OR = 0.103, 95% CI: 0.053–0.201) were barriers. For the high group, educational level (OR = 8.010, 95% CI: 1.855–34.597) and HPLP-II total score (OR = 1.147, 95% CI: 1.113–1.183) were facilitators, while alcohol consumption (OR = 0.101, 95% CI: 0.047–0.218) was a barrier.

**Conclusion:**

The total scores for health behavior protection and motivation, and the total HPLP-II scores, among UIA patients were generally moderate. Furthermore, health behavior protection motivation exhibited heterogeneity and could be classified into three distinct latent profiles. Clinically, personalized intervention plans can be developed based on the characteristics and latent profiles of patients, with the aim of enhancing patients’ levels of health behavior protection motivation, improving health-promoting behaviors, reducing the risk of aneurysm rupture, and improving long-term disease prognosis.

## 1 Introduction

An unruptured intracranial aneurysm is defined as an intracranial aneurysm with no history of rupture and bleeding or with a pathologically confirmed intact aneurysm wall. This category includes incidentally discovered aneurysms and unruptured aneurysms among multiple aneurysms (1). Among cerebrovascular events, the rupture of intracranial aneurysms (IA) is the third leading cause of morbidity and mortality from cerebrovascular disease, following ischemic stroke and hypertensive intracerebral hemorrhage (2). The risk of rupture is approximately 1.4% within 1 year and 3.4% within 5 years; once ruptured, the disability rate can reach 36–58% (3). Among surviving patients, 30–50% may subsequently experience varying degrees of neurological dysfunction or impairment in activities of daily living (4). Furthermore, once an IA ruptures, there is a 35% chance it will lead to aneurysmal subarachnoid hemorrhage (aSAH), leaving patients prone to persistent neurological deficits and impaired activities of daily living, resulting in a significant decline in their quality of life. This imposes a heavy disease burden on individuals, families, and society (5, 6).

The progression of IA is closely linked to health behaviors. Health behaviors (7) refer to a series of proactive, health-promoting actions individuals take to achieve better health, primarily including exercise, a balanced diet, actively seeking health information, regular health monitoring, adherence to medical advice, maintaining a positive attitude toward life, and stress management. Studies have shown that unhealthy behaviors such as smoking, heavy alcohol consumption, and staying up late may increase the risk of UIA rupture, whereas healthy behaviors such as regular exercise, maintaining a healthy weight, and managing diabetes and hypercholesterolemia may reduce the risk of UIA rupture (8). Furthermore, when patients with unruptured intracranial aneurysms (UIA) adopt proactive health behaviors, they can effectively reduce the incidence and recurrence of cerebrovascular events (9). Therefore, maintaining healthy behaviors among UIA patients, improving health literacy, and preventing UIA rupture at an early stage are crucial for reducing the risk of death. Protection Motivation Theory (PMT) is considered an effective theoretical framework for health education and has been repeatedly shown to facilitate the formation of health behaviors (10). According to PMT, health behavior is shaped by two cognitive appraisal processes: threat appraisal and coping appraisal (10). Threat appraisal consists of perceived severity, perceived susceptibility, and internal/external rewards, while coping appraisal comprises self-efficacy, response efficacy, and response costs. PMT posits that strengthening perceived severity, susceptibility, response efficacy, and self-efficacy, while reducing rewards and response costs, facilitates the formation of protection motivation and subsequent health-promoting behaviors (10). Therefore, this study, grounded in PMT, explains the formation of individual health behaviors from a motivational perspective and can be used to predict, interpret, and intervene in patients’ health behaviors with UIA.

As a long-term dynamic process, health behavior changes in response to disease progression and over time. Currently, international scholars agree on the necessity of long-term follow-up for UIA patients (11) and are gradually standardizing measures for UIA treatment, health monitoring, and behavioral management (12). In contrast, most domestic studies have adopted a “variable-centered” perspective, using scale scores to describe patients’ health behavior levels. These studies are limited to the external manifestations of health behavior and lack in-depth exploration of the intrinsic motivations behind UIA patients’ health behaviors. They also fail to address the heterogeneity of the UIA patient population, overlooking qualitative or quantitative differences in behavioral development among individuals (13, 14). Given the current lack of research on latent profile analysis of protection motivation levels among UIA patients both domestically and internationally, and considering that individual-centered analytical methods (such as latent profile analysis) can classify individuals into latent categories and determine the proportion of each category based on their scores on manifest variables, it is necessary to further investigate the characteristics of different latent profile groups to provide a reference for subsequent research on precision interventions targeting the health behaviors of UIA patients (15). LPA offers a distinct advantage over variable-centered approaches by identifying patient subgroups based on configurational patterns across all six PMT dimensions, rather than assuming population homogeneity. This is particularly important because identical total protection motivation scores may reflect qualitatively different dimensional combinations—for example, low self-efficacy may dominate in one subgroup, while high response costs predominate in another—each requiring fundamentally different intervention strategies. Such heterogeneity is inherently obscured in total-score or regression analyses, which treat dimensions as independent predictors. Thus, LPA provides an essential empirical basis for designing targeted, mechanism-driven interventions to optimize health behavior outcomes in UIA patients.

Against this backdrop, and in response to the current research gap regarding the latent profile analysis of health behavior protection motivation levels among UIA patients both domestically and internationally, this study aims to employ latent profile analysis to identify and characterize the latent profiles of health behavior protection motivation among UIA patients. It seeks to reveal the key factors influencing protection motivation levels across different profile types, thereby providing a scientific basis for developing targeted health interventions, enhancing patients’ self-management capabilities, and reducing the risk of aneurysm rupture.

## 2 Methods

### 2.1 Study population

Using convenience sampling, patients from the neurosurgery ward or the outpatient clinic of a Grade III-A hospital between July and December 2025 were selected as study subjects for the survey. Inclusion criteria: ① Unruptured intracranial aneurysm confirmed by head CT angiography or digital subtraction angiography; ② Normal cognitive and communication abilities, with the ability to clearly answer interview questions; ③ Willingness to participate in this study and signing of an informed consent form. Exclusion criteria: ① Patients with a history of severe neurological diseases such as cerebral infarction or cerebral hemorrhage; ② Patients with other severe comorbidities, such as significant cardiac, hepatic, renal, or pulmonary insufficiency; ③ Patients with psychiatric disorders, severe cognitive impairment, or motor dysfunction that would prevent them from independently completing the questionnaire or interview.

A total of 358 patients with UIA were initially assessed for eligibility at the neurosurgery ward and outpatient clinic of a Grade A tertiary hospital between July and December 2025. Of these, 32 were excluded: 12 had severe cognitive impairment or psychiatric disorders, 11 had severe cardiac, hepatic, or renal insufficiency, and 9 declined to participate. The remaining 326 patients completed the survey. After excluding 26 invalid responses (e.g., those with identical answers across all items or >20% missing data), 300 valid responses were included in the final analysis, yielding an effective response rate of 92.03%.

### 2.2 Sample size

According to Kendall’s principle (16), the sample size is generally 5 to 10 times the number of study variables. This survey included 9 variables on participants’ basic characteristics, 10 variables on disease-related factors, and 6 dimensions of the Health Behavior Protection Motivation Scale. Given a 20% ineligibility rate, the required sample size was at least 163. This study ultimately collected 300 participants to ensure the scientific validity and reliability of the research.

In addition to the sample size calculation based on Kendall’s principle, we further evaluated whether the final sample of 300 was adequate for the primary statistical methods used in this study. For latent profile analysis (LPA), methodological simulations suggest that a total sample size of 300 is sufficient for accurately recovering a three-class solution under moderate class separation (17, 18). Moreover, a minimum class size of 50 cases is recommended to ensure stable parameter estimates within each class (19). In our study, the smallest identified class (the low protection motivation group) contained 64 cases, accounting for 21% of the total sample, which exceeds the commonly recommended minimum threshold of 5% for class proportions (20). For the multinomial logistic regression analysis, we applied the “events per variable” (EPV) criterion. With seven independent variables included in the final model and 64 cases in the smallest outcome category (the low protection motivation group), the EPV ratio was approximately 9:1, slightly below the conventional recommended minimum of 10:1 (21). Nevertheless, the model showed acceptable fit and stability, and the results should be interpreted with appropriate caution.

### 2.3 Data collection

Data collection for this study was conducted face-to-face with patients. Prior to the survey, the research objectives were explained to the participants. After obtaining their informed consent, the questionnaires were distributed. Participants completed the questionnaires independently; researchers provided explanations for any questions raised, and the questionnaires were filled out and collected on the spot. The survey duration was flexibly adjusted according to patient requests, typically lasting 15–20 min. Throughout the data collection process, care was taken to protect participants’ privacy.

### 2.4 Research tools

#### 2.4.1 Demographic questionnaire

Designed by the researchers, this questionnaire included demographic characteristics such as gender, age, educational level, marital status, and average monthly household income, as well as clinical information including smoking status, alcohol consumption, sleep quality, aneurysm diameter, number of aneurysms, aneurysm location, treatment method, and the presence of other chronic comorbidities.

#### 2.4.2 Health-promoting lifestyle profile II (HPLP-II)

The Health-Promoting Lifestyle Profile (HPLP) was originally developed by Walker et al. (39) and subsequently revised by Pender et al. (40) to become the HPLP-II, in order to optimize its psychometric properties and adapt it to a broader range of research needs (22). Cao Wenjun et al. (41) conducted cross-cultural adaptation and validation of the scale’s validity and reliability, resulting in the current version. This scale is used to assess the level of health behaviors among study participants. It comprises six dimensions—self-actualization, sense of responsibility for health, physical exercise, nutrition, social support, and stress management—totaling 52 items. Each item is rated on a 1–4 scale, where 1 indicates “never,” 2 indicates “sometimes,” 3 indicates “often,” and 4 indicates “always.” The total score ranges from 52 to 208. Based on the total score, health behavior levels are categorized as Excellent (172–208 points), Good (132–171 points), Fair (92–131 points), and Poor (52–91 points); higher scores indicate better health behavior. In this study, the scale’s overall Cronbach’s α was 0.93, indicating good internal consistency.

#### 2.4.3 Health behavior protection motivation scale for patients with Unruptured intracranial aneurysms

The Health Behavior Protection Motivation Scale for Patients with UIA was developed by our research team based on the Protection Motivation Theory. The development process followed a rigorous four-step procedure: (1) item pool generation through literature review and semi-structured interviews; (2) expert consultation and pilot testing; (3) psychometric evaluation in 437 UIA patients; and (4) translation into English. The final scale comprises 6 dimensions and 25 items. Each item uses a 5-point Likert scale, ranging from “Strongly Disagree” to “Strongly Agree,” with scores ranging from 1 to 5. The total score ranges from 25 to 125. Among these, “Internal and External Rewards” and “Reaction Costs” are reverse-scored dimensions; during data processing, a scoring conversion method is applied to reverse their scores. If the original item is scored on a 5-point scale, the values are converted as follows: 1 → 5, 2 → 4, 3 → 3, 4 → 2, and 5 → 1.

In our validation study (manuscript under review), the scale demonstrated acceptable psychometric properties. Content validity was established through expert consultation (I-CVI: 0.813–1.000; S-CVI/Ave: 0.967). Exploratory factor analysis extracted six factors corresponding to the six PMT dimensions, with a cumulative variance explanation of 77.604%. Confirmatory factor analysis confirmed the six-factor structure with acceptable model fit (*χ*^2^/df = 1.649, RMSEA = 0.051, CFI = 0.953, TLI = 0.946). Convergent validity was supported by AVE values of 0.539–0.695 and CR values of 0.821–0.931. Discriminant validity was confirmed. The Cronbach’s α coefficient was 0.898 for the total scale and ranged from 0.819 to 0.872 for the six dimensions, and the split-half reliability was 0.899.

### 2.5 Ethical considerations

This study was approved by the Ethics Committee of the Second Affiliated Hospital of Nanchang University (Ethics Approval No.: O-Yiyan Lenshen [2025] No. (52)). Prior to the study’s commencement, the research objectives were explained to all participants, and all subjects voluntarily signed written informed consent forms. This study employed an anonymous survey format, inviting only individuals who were informed and consented to participate; all participants signed informed consent forms. To protect participants’ privacy, all collected data were properly stored in an anonymous format and kept strictly confidential.

### 2.6 Data analysis

In this study, statistical analysis was performed using SPSS 27.0 software. Quantitative data that followed a normal distribution were reported as mean ± standard deviation; otherwise, as median and interquartile range. Categorical data were presented as rates and proportions. Latent profile analysis was conducted using Mplus 8.3, with scores on the six dimensions of health behavior protection motivation among UIA patients serving as manifest variables. Based on the Akaike Information Criterion (AIC), the Bayesian Information Criterion (BIC), the adjusted Bayesian Information Criterion (aBIC), entropy,the Lo–Mendell–Rubin Likelihood Ratio Test (LMRT), and the Bootstrap Likelihood Ratio Test (BLRT). Smaller values of AIC, BIC, and aBIC indicate better model fit (23); the closer the entropy value (ranging from 0 to 1) is to 1, the higher the accuracy (24); the BLRT and LMRT were used to assess the fit of the x-class model versus the x-1-class model; a *p*-value < 0.05 indicates that the x-class model fits the data better than the x-1-class model; Depending on the data type, the chi-square test, Fisher’s exact test, and one-way analysis of variance (ANOVA) were used to compare differences in general demographic characteristics, disease-related information, and other variables among latent profiles of protective motivation for health behaviors in patients with UIA; an unordered multiclass logistic regression model was used to explore the predictive factors of latent profiles of protective motivation.

All *p*-values in this study are two-tailed, with a significance level of *α* = 0.05; *p* < 0.05 was considered statistically significant. Effect estimates are reported with 95% confidence intervals where applicable. Exact *p* values are reported; when the *p* value was less than 0.001, it is reported as *p* < 0.001.

## 3 Results

### 3.1 General demographic characteristics of patients with unruptured intracranial aneurysms

Among the study participants, 54% were female; 42% were under 50 years of age; 82% were married; 42% had a monthly income of 3,000–5,000 yuan; 71.3% had a solitary aneurysm; and 55.3% received conservative treatment. Detailed information is presented in Table 1.

**Table 1:** Sociodemographic and disease-related characteristics of participants (*n* = 300).

Variable	Category	Number of cases	Percentage (%)
Gender	Male	138	46.0
	Female	162	54.0
Age	<50	126	42.0
	50–60	95	31.7
	>60	79	26.3
Marital status	Single/Divorced/Widowed	54	18.0
	Married	246	82.0
Educational attainment	Junior high school or below	92	30.7
	High school/vocational school	104	34.7
	College or higher	104	34.7
Average monthly income (yuan)	<3,000	84	28.0
	3,000–5,000	126	42.0
	>5,000	90	30.0
Number of aneurysms	Solitary	214	71.3
	Multiple	86	28.7
Aneurysm diameter (mm)	<5	124	41.3
	5–10	132	44.0
	>10	44	14.7
Treatment approach	Conservative treatment	166	55.3
	Surgical treatment	134	44.7
Smoking	Yes	85	28.3
	No	215	71.7
Alcohol consumption	Yes	99	33.0
	No	201	67.0
Sleep	Poor	79	26.3
	Average	124	41.3
	Good	97	32.3
Comorbidities	Yes	116	38.7
	None	184	61.3

### 3.2 Level of protection motivation for health behaviors in patients with unruptured intracranial aneurysms

The results showed that the total protection motivation score in UIA patients was 81.96 ± 15.41. The total score for health behavior protection motivation, the total scores for each dimension, and the mean scores for each dimension are presented in Table 2. Overall, the findings indicate that the level of health behavior protection motivation among UIA patients is moderate, with variations in scores across dimensions, suggesting that latent profile analysis could be further employed to identify subgroups of patients with distinct motivational characteristics.

**Table 2:** Dimension scores of health behavior protection motivation in UIA patients (*N* = 300).

Dimension	Total score of each dimension (mean ± standard deviation)	Mean score of each dimension (mean ± standard deviation)
Severity	21.53 ± 5.08	3.58 ± 0.85
Susceptibility	10.60 ± 2.63	3.53 ± 0.88
Internal and external rewards	6.97 ± 3.18	2.32 ± 1.06
Reaction efficiency	13.03 ± 2.15	3.26 ± 0.54
Reaction cost	9.39 ± 1.68	3.13 ± 0.56
Self-efficacy	20.44 ± 2.48	3.41 ± 0.41
Total score	81.96 ± 15.41	

### 3.3 Current status of health-promoting lifestyle levels in patients with unruptured intracranial aneurysms

In this study, the Health-Promoting Lifestyle Profile II (HPLP-II) was used to assess the level of health-promoting behaviors in patients with unruptured intracranial aneurysms. The results showed a total HPLP-II score of 126.83 ± 30.78, indicating an overall moderate level. Detailed results are presented in Table 3.

**Table 3:** Total HPLP-II score and dimension scores in UIA patients (*N* = 300).

Dimension	Score (mean ± standard deviation)
Self-actualization	25.61 ± 5.94
Health responsibility	21.94 ± 5.46
Physical exercise	18.11 ± 4.66
Nutrition	23.80 ± 5.67
Social support	24.23 ± 5.75
Stress management	13.15 ± 3.61
Total score	126.83 ± 30.78

### 3.4 Correlation analysis between health behavior protection motivation and health behavior promotion in patients with unruptured intracranial aneurysms

Pearson correlation analysis was performed between the dimension scores of health behavior protection motivation and the total score of health behavior promotion (HPLP-II) in UIA patients. The results showed that the total protection motivation score was positively correlated with the total HPLP-II score (*r* = 0.733, CI: 0.675–0.781, *p* < 0.001), and the total scores of each protection motivation dimension were also positively correlated with the total health behavior promotion score (*p* < 0.001). Among all dimensions, self-efficacy showed the strongest correlation with the level of health behavior promotion (*r* = 0.752, CI: 0.698–0.797, *p* < 0.001). Additional correlation details are presented in Table 4.

**Table 4:** Correlation analysis between health behavior protection motivation and health behavior promotion in UIA patients.

Variable	r value for the total HPLP II score	*95% CI*	*p*
Severity	0.688	0.622–0.743	<0.001
Susceptibility	0.671	0.603–0.728	<0.001
Internal and external rewards	0.624	0.549–0.688	<0.001
Reaction efficiency	0.657	0.587–0.716	<0.001
Reaction cost	0.463	0.369–0.547	<0.001
Self-efficacy	0.752	0.698–0.797	<0.001
Total protection motivation score	0.733	0.675–0.781	<0.001

### 3.5 Latent profile analysis of health behavior protection motivation in patients with unruptured intracranial aneurysms

Latent profile analysis (LPA) was conducted using the six dimensions of the Protection Motivation Scale—namely, severity, susceptibility, internal and external rewards, response efficacy, response cost, and self-efficacy—as external indicators. Models with one to five latent profiles were fitted, and the fit indices were compared, including log-likelihood (LL), Akaike information criterion (AIC), Bayesian information criterion (BIC), sample-size adjusted Bayesian information criterion (SABIC), and entropy. The optimal model was determined by evaluating these fit indices alongside class sample sizes and interpretability. Detailed results are presented in Table 5.

**Table 5:** Fit indices comparison of latent profile models for health behavior protection motivation in UIA patients (*K* = 1–5).

Number of classes (K)	AIC	BIC	aBIC	Entropy	LMR P	BLRT P	Class probabilities
1	3732.585	3777.030	3738.973	**—**	**—**	**—**	**—**
2	2748.653	2819.024	2758.068	0.882	**—**	**—**	**—**
**3**	**1712.452**	**1808.750**	**1726.293**	**0.971**	**0.0042**	**<0.001**	**0.21/0.53/0.26**
4	1228.795	1351.020	1246.363	0.975	0.0009	<0.001	0.21/0.20/0.37/0.22
5	963.024	1111.175	984.319	0.983	0.0002	<0.001	0.17/0.04/0.37/0.20/0.22

K is the number of freely estimated parameters; AIC is the Akaike Information Criterion; BIC is the Bayesian Information Criterion; aBIC is the sample-corrected Bayesian Information Criterion; Entropy is information entropy; LMR is the likelihood ratio test; BLRT is the bootstrap-based likelihood ratio test. Bold values indicate statistical significance.

The results show that as the number of latent profiles increases, AIC, BIC, and aBIC generally decrease, suggesting continuous improvement in model fit. However, model selection cannot rely solely on statistical metrics; it must also comprehensively consider category size, classification quality, and theoretical interpretability. Although the 4-category model outperformed the 3-category model in fit metrics, its newly added category primarily represented a further subdivision of the intermediate-level population, with limited improvement in distinguishing clinical characteristics between categories; meanwhile, while some fit metrics continued to decline in the 5-category model, small categories emerged. In contrast, the 3-class model exhibits high entropy, good classification accuracy, a reasonable distribution of samples across classes, no underrepresented classes, and clear class characteristics. It demonstrates a gradient distribution of low–medium–high health behavior protection motivation, offering good theoretical interpretability and clinical applicability. Therefore, after comprehensively evaluating fit indices, class size, and theoretical interpretability, this study ultimately selected the 3-class latent profile model for subsequent analysis.

Further calculations of the latent profile membership probabilities showed that the average membership probabilities for samples in each category were high across all three latent profile models. The diagonal probabilities for the low-protection, medium-protection, and high-protection groups were 1.000, 0.991, and 0.955, respectively—all above 0.80—while the off-diagonal probabilities were relatively low, indicating good classification accuracy and stability. The average posterior probability matrix for the three classes is presented in Table 6.

**Table 6:** Average posterior probabilities for each latent profile of health behavior protection motivation in UIA patients.

Latent profile	Number of cases	Percentage (%)	Average posterior probability		
			C1(%)	C2(%)	C3(%)
C1	64	0.21	1.000	0.000	0.000
C2	158	0.53	0.000	0.991	0.009
C3	78	0.26	0.000	0.045	0.955

C1, Low-protection group; C2, Medium-protection group; C3, High-protection group.

The results showed that the different latent profiles differed significantly in scores across all dimensions of protection motivation. The conditional means of the three classes of health behavior protection motivation in UIA patients across the six dimensions are presented in Table 7 and Figure 1. Based on the score characteristics across dimensions and group performance, the three classes were named as follows:

**Table 7:** Comparison of mean dimension scores of protection motivation across different latent profiles in UIA patients.

Variable	Class 1 (*n* = 64)	Class 2 (*n* = 158)	Class 3 (*n* = 78)
Susceptibility	2.099	3.783	4.434
Severity	2.131	3.622	4.532
Internal and external rewards	2.933	2.866	3.852
Reaction cost	1.390	2.191	3.392
Reaction efficiency	2.730	3.138	3.956
Self-efficacy	3.005	3.297	3.977

**Figure 1:**
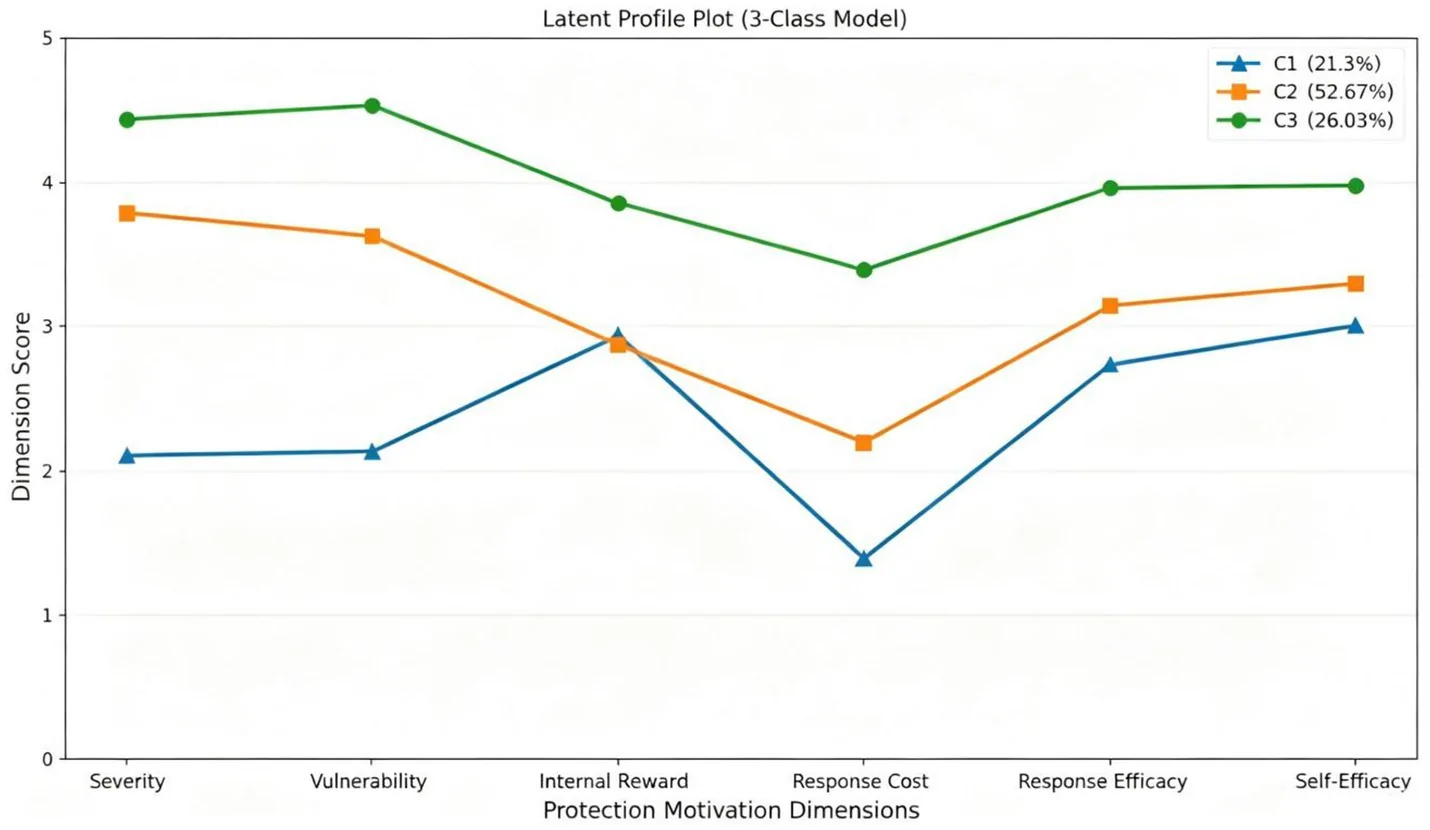
Latent profile characteristics of health behavior protection motivation in UIA patients.

Category 1 comprises 64 patients (21%). Their mean scores across all items in each dimension are low, indicating that these patients generally have low motivation for health-protective behaviors, low recognition of their effectiveness, and low confidence in their ability to adopt them. Therefore, this group is named the “Low Protection Motivation Group.”

Category 2 comprises 158 patients (53%), whose mean scores across all dimension items are moderate. This suggests that these patients possess a certain level of awareness of disease risks, recognize the positive effects of health-protective behaviors to some extent, and have a degree of confidence in their ability to implement them. Therefore, this group is named the “Moderate Protection Motivation Group.”

Category 3 comprises 78 patients (26%), whose mean scores across all dimension items are high. This indicates that these patients have a strong awareness of the potential risks and serious consequences of UIA, fully recognize the effectiveness of health-protective behaviors, and maintain a high level of confidence in their ability to adopt such behaviors. Therefore, this group is designated as the “High Motivation Group.”

Overall, the three potential profiles exhibited distinct gradients in scores across the various dimensions of protective motivation, indicating significant heterogeneity in protective motivation for health behaviors among patients with unruptured intracranial aneurysms.

### 3.6 Univariate analysis of latent profiles of health behavior protection motivation in patients with unruptured intracranial aneurysms

To explore differences in general demographic and disease-related characteristics among patients in different latent profiles, this study conducted univariate analyses of general demographic and disease-related data, using latent profile categories as grouping variables. Categorical variables were analyzed using the chi-square (*χ*^2^) test, and the results are presented in Table 8. The results showed statistically significant differences (*p* < 0.05) among patients with different latent profiles in terms of educational level, comorbid chronic diseases, sleep, smoking, alcohol consumption, and the HPLP II total score. Other clinical variables, including aneurysm diameter, number of aneurysms, treatment approach, and time since diagnosis, showed no statistically significant differences across the three latent profiles (*p* > 0.05 for all) and were therefore not entered into the Multinomial logistic regression model.

**Table 8:** Univariate analysis of factors associated with different latent profiles in UIA patients.

Variable	Low protection motivation group	Moderate protection motivation group	High protection motivation group	*χ*^2^/*F*	*p*
Gender *n* (%)				0.320	0.984
Female	34 (53.1%)	86 (54.4%)	42 (53.8%)		
Male	30 (46.9%)	72 (45.6%)	36 (46.2%)		
Age				4.242	0.374
<50 years old	33(51.6%)	66(41.8%)	27(34.6%)		
50–60 years old	17(26.6%)	51(32.3%)	27(34.6%)		
Over 60	14(21.9%)	41(25.9%)	24(30.8%)		
Marital status				0.620	0.734
Married	54(84.4%)	127(80.4%)	65 (83.3%)		
Unmarried/Divorced	10 (15.6%)	31 (19.6%)	13 (16.7%)		
Educational attainment				19.175	<0.001
Junior high school or below	32 (50.0%)	43(27.2%)	17 (21.8%)		
High school/vocational school	22 (34.4%)	53(33.5%)	29 (37.2%)		
College or higher	10(15.6%)	62 (39.2%)	32 (41.0%)		
Household income				4.381	0.305
<3,000 yuan	23(35.9%)	40(25.3%)	21(26.9%)		
3,000–6,000 yuan	27(42.2%)	70(44.3%)	29(37.2%)		
>6,000 yuan	14(21.9%)	48(30.4%)	28(35.9%)		
Number of aneurysms				0.110	0.946
Solitary	45(70.3%)	114 (72.2%)	55(70.5%)		
Multiple	19 (29.7%)	44 (27.8%)	23(29.5%)		
Aneurysm diameter groups (mm)				3.279	0.512
<5	30 (46.9%)	67(42.4%)	27(34.6%)		
5–10	27 (42.2%)	69(43.7%)	36(46.2%)		
>10	7(10.9%)	22(13.9%)	15(19.2%)		
Smoking				7.693	0.021
Yes	27(42.2%)	39(24.7%)	19(24.4%)		
No	37 (57.8%)	119(75.3%)	59(75.6%)		
Alcohol consumption				45.925	<0.001
Yes	47 (73.4%)	35 (22.2%)	17 (21.8%)		
No	17(26.6%)	123 (77.8%)	61 (78.2%)		
Sleep quality				60.171	<0.001
Fair	24 (37.5%)	62 (39.2%)	38 (48.7%)		
Good	10 (15.6%)	71 (44.9%)	16 (20.5%)		
Poor	30 (46.9%)	25 (15.8%)	24 (30.8%)		
Coexisting chronic conditions				11.486	0.003
None	39 (60.9%)	109 (69.0%)	36 (46.2%)		
Yes	25 (39.1%)	49 (31.0%)	42 (53.8%)		
Time of diagnosis				4.467	0.346
<1 year	29 (45.3%)	57 (36.1%)	28 (35.9%)		
1–2 years	12 (18.8%)	43 (27.2%)	26 (33.3%)		
≥2 years	23 (35.9%)	58 (36.7%)	24 (30.8%)		
HPLP II total score (x̄±s)	94.78 ± 18.09	125.08 ± 22.31	156.67 ± 25.20	136.459	**<0.001**

Data are presented as *n* (%). *χ*^2^tests were used for categorical variables; one-way ANOVA was used for continuous variables (HPLP-II total score). Exact p values are reported; *p* < 0.001 is reported for values below this threshold. HPLP-II, Health-Promoting Lifestyle Profile II. Bold values indicate statistical significance.

### 3.7 Multinomial analysis of latent profiles of health behavior protection motivation in patients with unruptured intracranial aneurysms

Using latent profiles of health behavior protection motivation in patients with unruptured intracranial aneurysms as the dependent variable, with the low protection motivation group serving as the reference group, and incorporating items that showed statistically significant differences in univariate analysis as independent variables, we constructed an unordered multiclass logistic regression model. The assignment of independent variables is detailed in Table 9. The results show that, compared with the low-protection group, having a college degree or higher, good sleep quality, and a higher HPLP-II total score were factors promoting assignment to the moderate-protection group, while smoking and alcohol consumption were inhibiting factors; having a college degree or higher and a higher HPLP-II total score were factors promoting assignment to the high-protection group, while alcohol consumption was an inhibiting factor as shown in Table 10.

**Table 9:** Variable coding for multinomial analysis.

Variable name	Coding
Gender	Male = 1, Female = 0
Educational level	Junior high school or below = 2, high school/vocational school = 1, college or higher = 0
Sleep	Good = 2, Fair = 1, Poor = 0
Coexisting chronic diseases	Yes = 1, No = 0
Smoking	Yes = 1, No = 0
Alcohol consumption	Yes = 1, No = 0
HPLP II	Substitute raw scores

**Table 10:** Logistic regression analysis of latent profiles of health behavior protection motivation in patients with UIA.

Group	Variable name	OR	95% CI	*p*
Moderate protection motivation group	Educational level	6.997	2.053 ~ 23.850	0.002**
	Sleep	5.069	1.644 ~ 15.626	0.005**
	Smoking	0.357	0.138 ~ 0.928	0.035*
	Alcohol Consumption	0.103	0.053 ~ 0.201	<0.001***
	HPLP II total score	1.078	1.051 ~ 1.105	<0.001***
High protection motivation group	Educational level	8.010	1.855 ~ 34.597	0.005**
	Alcohol consumption	0.101	0.047 ~ 0.218	<0.001***
	HPLP II total score	1.147	1.113 ~ 1.183	<0.001***

Model fit statistics: −2 Log Likelihood = 326.105; Model *χ*^2^ = 280.818, df = 16, *p* < 0.001; Cox & Snell R^2^ = 0.608; Nagelkerke *R*^2^ = 0.699; McFadden *R*^2^ = 0.460.

All covariates listed in Table 9 were entered into the multinomial logistic regression model; only statistically significant variables were presented in this table. *p < 0.05, **p < 0.01, ***p < 0.001. Reference category = Low protection motivation group.

## 4 Discussion

### 4.1 Current status of health behavior protection motivation among UIA patients

The results of this study indicate that the overall score for health behavior protection motivation among UIA patients is moderate. This suggests that while most patients possess a certain level of disease risk perception and willingness to change their health behaviors, there is still significant room for improvement in their overall protection motivation. For some UIA patients, although they clearly face disease-related risks, whether they can further translate this risk perception into sustained, stable protective behaviors in their daily health management remains influenced by a combination of various factors. This suggests that, in addition to focusing on the disease itself, clinical nursing staff should also prioritize stimulating and maintaining patients’ motivation for healthy behaviors during routine diagnosis and treatment (25).

Looking at the scores across each dimension, the Severity and Susceptibility dimensions scored relatively high, suggesting that UIA patients have some awareness of the serious consequences of the disease and their own risk of developing it. This may be related to the health education provided by healthcare professionals to patients after diagnosis, and it also reflects that the diagnosis itself can, to some extent, heighten patients’ perception of threat.

Scores on the response efficacy and self-efficacy dimensions were in the upper-middle range, indicating that patients generally recognize the effectiveness of health behavior interventions and have a certain level of confidence in their ability to carry out health management behaviors. However, since these scores did not reach a high level, this suggests that while some patients are aware of the benefits of healthy behaviors, they still lack confidence and face challenges in actual implementation. This suggests that clinicians should use health education, behavioral guidance, and ongoing follow-up to enhance patients’ awareness of the benefits of healthy behaviors and their self-management capabilities.

The total scores for the Internal and External Rewards and Response Cost dimensions were among the lowest of all dimensions. This suggests that patients perceive certain costs and barriers when faced with changes in health behaviors, such as the inconvenience caused by lifestyle changes, the difficulty of long-term adherence, and increased time and energy requirements. These results indicate that although UIA patients possess a certain level of health risk awareness, if intervention measures remain limited to risk communication without providing concrete, actionable behavioral support, patients may struggle to truly translate their protective motivation into sustained health behaviors.

In summary, in clinical nursing practice, merely conveying disease knowledge to UIA patients is insufficient;but efforts should also be made to help patients recognize the long-term benefits of healthy behaviors and reduce their subjective perception of the costs associated with behavioral change,such as by setting realistic and achievable small goals, strengthening family support, and providing more practical behavioral plans, thereby truly enhancing patients’ levels of protection motivation (26).

### 4.2 Current status of health behavior promotion levels in UIA patients

The results of this study show that the overall HPLP-II total score for UIA patients is moderate. This finding is consistent with the results of a study by Lee et al. (27). Looking at scores across each dimension, self-actualization, interpersonal support, and nutrition were relatively high, suggesting that patients have a certain foundation in self-awareness, interpersonal interaction, and dietary regulation. In contrast, scores for physical exercise and stress management were relatively low, suggesting that patients still face challenges in maintaining long-term physical activity, managing emotions, and regulating their daily routines. These findings align with general patterns of health behaviors among patients with chronic diseases: patients tend to be more receptive to well-defined behaviors such as dietary control, outpatient follow-ups, and medication adherence, while adherence is typically lower for behaviors requiring long-term commitment and involving significant individual variation in subjective experience, such as exercise, stress management, and sleep improvement (28).

For patients with UIA, health behavior promotion is not limited to a healthy lifestyle but is also closely related to cardiovascular and cerebrovascular risk management. Therefore, clinical nursing interventions should place greater emphasis on linking health behavior promotion to disease risk management, helping patients understand that these lifestyle adjustments are not merely general health education but specific protective measures closely related to the risk of aneurysm rupture (29).

### 4.3 A correlation exists between health behavior protection motivation and health behavior promotion levels in UIA patients

The results of this study show that the total score for health behavior protection motivation among UIA patients is significantly positively correlated with the total score for health behavior promotion (*r* = 0.733, CI: 0.675–0.781, *p* < 0.001), and the total scores for each dimension of protection motivation are also positively correlated with the total score for health behavior promotion (*p* < 0.001). This suggests that health behavior protection motivation is positively associated with health behavior promotion among UIA patients. These findings are consistent with those reported by Tu et al. (30). This indicates that protective motivation plays a crucial role in the formation and maintenance of health behaviors among UIA patients; patients are more likely to proactively adopt and adhere to health-promoting behaviors only when they fully recognize the risks of their condition, acknowledge the value of healthy behaviors, and possess the corresponding behavioral confidence.

Among these factors, self-efficacy showed the strongest correlation with the level of health-promoting behavior, suggesting that self-efficacy is a key factor influencing patients’ ability to translate health-related knowledge into actual behavior. Even if patients are aware that unhealthy behaviors may increase the risk of aneurysm rupture, they will still find it difficult to consistently adopt health-promoting behaviors if they lack a sufficient sense of behavioral control and confidence in their ability to persist. This finding is consistent with protection motivation theory and suggests that self-efficacy may be a key area for emphasis in clinical interventions.

In summary, there is a close association between patients’ protective motivation for health behaviors and their level of health behavior promotion among those with UIA; the stronger the protective motivation, the more likely patients are to adopt positive health behaviors. These findings suggest that interventions targeting protective motivation may be beneficial for improving health behaviors among UIA patients. However, due to the cross-sectional design, causal direction cannot be established, and future longitudinal or interventional studies are needed to confirm this relationship.

### 4.4 Population heterogeneity in health behavior protection motivation among UIA patients

Through latent profile analysis, this study identified three patient groups with different levels of health behavior protection motivation: the low protection motivation group, the moderate protection motivation group, and the high protection motivation group. The scores of these three groups across the six dimensions of protection motivation showed a distinct gradient distribution, suggesting that protection motivation for health behavior among UIA patients exhibits group heterogeneity.

An analysis of dimension scores across the different profiles reveals that the areas of weakness in protective motivation vary among patient groups, indicating that this heterogeneity is reflected not only in overall motivation levels but also in differences in the specific structural composition of the dimensions. In the low protective motivation group, the score for “reaction costs” was the lowest, followed by “vulnerability” and “severity.” This suggests that patients in this group experience greater difficulty and burden when engaging in health behaviors, while simultaneously having a relatively insufficient understanding of disease risks and severe consequences. This reflects that their weak protective motivation is related not only to insufficient threat perception but also to a strong sense of the costs associated with behavioral change. It is worth noting that the low-protective-motivation group scored significantly higher on the internal and external reward dimensions than the moderate-protective-motivation group. This trend is inconsistent with the gradient distribution observed in other dimensions, suggesting that patients in this group exhibit unique maladaptive characteristics in their cognitive structure. Patients in the low protective motivation group may be more inclined to derive immediate satisfaction from current unhealthy but comfortable behaviors—that is, they have a stronger perception of internal rewards. At the same time, their external support environment may lack sufficient supervision or risk warnings; external feedback may even unintentionally reinforce unhealthy behaviors, leading them to fail to perceive external pressure to change their behavior. From a behavioral economics perspective, this group of patients exhibits distinct “present bias,” meaning they prioritize short-term convenience and comfort over the health benefits of delayed gratification (31). This suggests that weak protective motivation is not only manifested as insufficient risk perception or low self-efficacy but may also stem from a subjective overestimation of the rewards associated with unhealthy behaviors. In the moderate protective motivation group, response costs remained the lowest dimension, followed by internal and external rewards. This suggests that although patients in this group possess a certain level of risk awareness, they are relatively sensitive to the potential costs involved in behavioral change and still have a relatively limited perception of the benefits of healthy behaviors; therefore, their protective motivation has not yet been consistently translated into a higher level of behavioral readiness. In the high protective motivation group, response costs were the relatively lowest dimension, followed by internal and external rewards, indicating that even among individuals with overall high protective motivation, the costs of behavioral implementation remain a key factor limiting the further reinforcement and long-term maintenance of healthy behaviors.

These results suggest that clinical interventions should implement stratified management targeting the weak dimensions of different potential profiles: for the group with low protective motivation, the focus should be on strengthening disease risk awareness and reducing resistance to behavioral change; given their “present bias,” it is necessary to move beyond mere risk warnings and instead use motivational interviewing to encourage them to re-evaluate the trade-off between short-term convenience and long-term costs, while using small-goal check-ins to enhance immediate positive feedback;optimizing social support, providing concurrent education for family members to reduce the influence of external rewards, and highlighting the tangible benefits of healthy behaviors (32) to counteract the present bias caused by high internal and external rewards (33); For the moderate protection motivation group, the focus should be on enhancing the perception of the benefits of healthy behaviors, helping them translate existing cognition into sustained behavior; for the high protection motivation group, attention should be directed toward reducing the practical burdens associated with behavioral persistence to promote the long-term maintenance of healthy behaviors. This approach is more effective at enhancing overall health behavior protection motivation among UIA patients (26).

### 4.5 Analysis of factors influencing latent profiles of health behavior protection motivation in UIA patients

This study found that multiple demographic, clinical, and behavioral factors are significant predictors of latent profile grouping for protection motivation in UIA patients. A higher total HPLP-II score is a common contributing factor for patients being classified into the moderate- and high-protection-motivation groups. This indicates that patients with higher levels of health behavior promotion are more likely to develop high levels of protection motivation, suggesting a mutually reinforcing relationship between health behaviors and protection motivation (34). Educational attainment is also an important predictor; patients with higher levels of education are more likely to be classified into the moderate and high protection motivation groups. This is because individuals with higher educational attainment have advantages in understanding disease information, acquiring health knowledge, and communicating with healthcare providers, enabling them to better recognize the intrinsic link between health behaviors and disease prognosis (35, 36). Conversely, smoking and alcohol consumption act as negative barriers: smoking hinders patients from entering the moderate protection motivation group, while alcohol consumption was associated with a lower likelihood of being classified into the moderate protection motivation group and the high protection motivation group. Descriptively, 73.4% of patients in the low protection motivation group reported alcohol consumption, compared with 22.2% in the moderate group and 21.8% in the high group, indicating that drinkers were less likely to develop higher levels of protection motivation than non-drinkers. This suggests that long-term unhealthy lifestyle habits, psychological dependence, and sociocultural factors can weaken patients’ willingness and ability to change their behavior; even when patients are aware that such habits increase the risk of poor outcomes for intracranial aneurysms, they find it difficult to make behavioral adjustments (37). Sleep quality also plays a significant role; patients with poor sleep are more likely to be classified as low-protection. Chronic sleep disturbances are closely associated with fatigue, anxiety, reduced self-regulation, and impaired behavioral execution (38); all of these factors can hinder the formation and maintenance of health-protective motivation. Furthermore, univariate analysis results indicate that the presence of chronic comorbidities is statistically significant. The reason for this is that patients with prior experience managing chronic diseases have a deeper understanding of the importance of healthy behaviors and are more proficient in areas such as regular medication adherence, scheduled follow-up visits, dietary control, and self-health monitoring; consequently, they demonstrate greater acceptance of and ability to implement health-protective behaviors.

In summary, this study’s results suggest that clinical interventions for patients with unruptured intracranial aneurysms should adopt a stratified, individualized approach. Targeted intervention plans should be developed by comprehensively considering patients’ educational background, lifestyle (particularly smoking, alcohol consumption, and sleep patterns), history of chronic diseases, and baseline health-promoting behaviors; these plans should combine enhancing health-protective motivation with comprehensive health behavior promotion to establish an integrated intervention system. At the same time, special attention should be given to patients with lower educational attainment by adopting health education formats that are easy to understand—such as educational graphics, short videos, scenario-based demonstrations, and family-collaborative health education—to improve the accessibility and effectiveness of health education.

It is important to note that the HPLP-II total score and protection motivation are conceptually interrelated, as protection motivation is theorized to be a proximal determinant of health-promoting behaviors. In this study, both constructs were measured at the same time point, and the observed correlation was substantial (*r* = 0.73, *p* < 0.001). Therefore, the finding that a higher HPLP-II total score predicted membership in the moderate and high protection motivation groups should be interpreted with caution. This association likely reflects a mutually reinforcing relationship between health behaviors and protection motivation rather than a unidirectional causal effect. Individuals who engage in more health-promoting behaviors may develop stronger confidence and motivation to maintain those behaviors, while those with higher protection motivation are more likely to adopt and sustain healthy behaviors. Due to the cross-sectional design, the direction of this relationship cannot be determined. Future longitudinal studies are needed to clarify the temporal sequence and potential reciprocal dynamics between protection motivation and health behaviors in UIA patients.

### 4.6 Common method bias

Both protection motivation and health-promoting behaviors were assessed using self-report questionnaires, which may introduce common method bias. This could potentially inflate the observed correlation between the two constructs (*r* = 0.73, *p* < 0.001). Although we employed several procedural remedies—including anonymous responses, standardized instructions, and ensuring item clarity during data collection—to minimize this bias, it cannot be entirely ruled out. Future studies should incorporate objective measures to validate the relationship between protection motivation and health behaviors in UIA patients.

## 5 Limitations

Although this study revealed the overall level and dimensional characteristics of health behavior protection motivation among UIA patients and analyzed its potential profiles and influencing factors, the following limitations remain: First, the sample was drawn from a single Grade A tertiary hospital, which may affect the representativeness of the results; Additionally, both protection motivation and health-promoting behaviors were measured via self-report questionnaires, which may introduce common method bias and potentially inflate the observed correlation; future studies should incorporate objective measures to validate these findings. Second, the cross-sectional design makes it difficult to infer causal relationships; Third, while this study selected the optimal model—comprising three profiles—by balancing statistical indicators and clinical utility, it should be acknowledged that the four-category model also performed well in terms of fit indices (AIC, BIC, aBIC), suggesting that the heterogeneity of protective motivation may involve more refined subtypes; the current classification scheme requires further external validation; Fourth, no intervention studies have yet been conducted to verify the predictive validity and clinical efficacy of the scale. Recommendations for the Future: Conduct longitudinal studies to explore the trajectories of protective motivation over time and its impact on health outcomes; expand the sample size and include more clinical characteristic variables to further explore the stability and external validity of the four-category model—or even more categories—to verify whether subtypes with greater clinical discriminative value exist; design and implement differentiated intervention programs based on the profile classification to verify their effectiveness in improving patients’ health behaviors and clinical outcomes.

## 6 Conclusion

Using latent profile analysis, this study identified three latent profiles of health behavior protection motivation among patients with unruptured intracranial aneurysms: low, moderate, and high. The total score for health behavior promotion is a key determinant of the level of health behavior promotion. Healthcare professionals should implement targeted health interventions based on these categories, with a particular focus on high-risk populations, such as those with low educational attainment, poor sleep quality, smoking, alcohol consumption, or comorbid chronic diseases. Future research could further investigate the dynamic changes and underlying mechanisms of protective motivation across these categories, develop long-term intervention plans tailored to patients’ individual characteristics, and explore diverse health education models to continuously optimize health management strategies for patients with unruptured intracranial aneurysms. In summary, higher levels of protective motivation were associated with better health behaviors in patients with unruptured intracranial aneurysms. Interventions aimed at enhancing protective motivation may help patients adhere to health management requirements and improve their long-term health status; however, longitudinal studies are needed to confirm the causal direction of this relationship.

## Data Availability

The data analyzed in this study is subject to the following licenses/restrictions: The authors will provide all original data relevant to this study without reservation. It is available by contacting the corresponding author for valid reasons. Requests to access these datasets should be directed to CZ, 931048874@qq.com.
