# Supplementary material for: Analysis of the potential profile and influencing factors of health behavior protection motivation in patients with unruptured intracranial aneurysms based on protection motivation theory
Source: Front Public Health. 2026 Oct 14;14:1942155. doi: 10.3389/fpubh.2026.1942155 (PMC13713687; doi:10.3389/fpubh.2026.1942155)
Supplement: Supplementary file 1 [file Table_1.DOCX]

Supplementary Material

**Appendix: “Assessment Scale for Health Behavior Protection Motivation in Unruptured Intracranial Aneurysms”**

Instructions: This questionnaire assesses your current level of motivation regarding health-promoting behaviors. Please answer all questions to the best of your ability and select the option that best reflects your view.

Strongly Disagree: I completely disagree with this statement.

Disagree: I moderately disagree with this statement.

Neutral:I have no particular opinion on this statement; I neither agree nor disagree.

Agree:I moderately agree with this statement.

Strongly Agree: I completely agree with this statement.

| **Items** | **Strongly Disagree** | **Disagree** | **Neutral** | **Agree** | **Strongly Agree** |
| --- | --- | --- | --- | --- | --- |
| 1. If I don’t quit smoking and drinking, my risk of an aneurysm rupture will increase. |  |  |  |  |  |
| 2. Insufficient and irregular sleep will increase my risk of an aneurysm rupture. |  |  |  |  |  |
| 3. Failure to take my medications on schedule will increase my risk of an aneurysm rupture. |  |  |  |  |  |
| 4. Failure to give up high-fat and high-salt diets will increase my risk of aneurysm rupture. |  |  |  |  |  |
| 5. Emotional instability will increase my risk of aneurysm rupture. |  |  |  |  |  |
| 6. Lack of regular, moderate physical activity will increase my risk of aneurysm rupture. |  |  |  |  |  |
| 7. An uncontrolled, unruptured intracranial aneurysm may rupture. |  |  |  |  |  |
| 8. Aneurysm rupture poses a life-threatening risk to me. |  |  |  |  |  |
| 9. Aneurysm rupture will increase my financial burden. |  |  |  |  |  |
| 10. Smoking and drinking facilitate social interactions. |  |  |  |  |  |
| 11. Little or no physical activity makes me feel comfortable. |  |  |  |  |  |
| 12. My family and friends claim that an intracranial aneurysm is a minor condition that does not require frequent outpatient follow-ups. |  |  |  |  |  |
| 13. I believe dietary management helps prevent aneurysm rupture. |  |  |  |  |  |
| 14. I believe that taking medication regularly helps prevent aneurysm rupture. |  |  |  |  |  |
| 15. I believe maintaining a positive outlook helps prevent aneurysm rupture. |  |  |  |  |  |
| 16. I believe that regular, moderate physical exercise helps prevent aneurysm rupture. |  |  |  |  |  |
| 17. Regular blood pressure monitoring is a hassle for me. |  |  |  |  |  |
| 18. Healthy eating habits take away from the pleasure of enjoying delicious food. |  |  |  |  |  |
| 19. Maintaining a regular, healthy sleep schedule takes up my free time at night. |  |  |  |  |  |
| 20. I am confident that I can stick to a regular, moderate exercise routine. |  |  |  |  |  |
| 21. I am confident that I can monitor my blood pressure and take my medications as prescribed, even when I’m out and about. |  |  |  |  |  |
| 22. I am confident that I can maintain stable, positive emotions. |  |  |  |  |  |
| 23. I am confident that I can follow a low-salt, low-fat diet. |  |  |  |  |  |
| 24. I am confident that I can quit smoking and limit my alcohol intake. |  |  |  |  |  |
| 25. I am confident that I can maintain a regular sleep schedule and avoid staying up late. |  |  |  |  |  |
